# Deep-Phenotyping the Less Severe Spectrum of *PIGT* Deficiency and Linking the Gene to Myoclonic Atonic Seizures

**DOI:** 10.3389/fgene.2021.663643

**Published:** 2021-05-11

**Authors:** Allan Bayat, Manuela Pendziwiat, Ewa Obersztyn, Paula Goldenberg, Pia Zacher, Jan Henje Döring, Steffen Syrbe, Amber Begtrup, Artem Borovikov, Artem Sharkov, Aneta Karasińska, Maria Giżewska, Wendy Mitchell, Eva Morava, Rikke S. Møller, Guido Rubboli

**Affiliations:** ^1^Institute for Regional Health Services, University of Southern Denmark, Odense, Denmark; ^2^Department of Epilepsy Genetics and Personalized Medicine, Danish Epilepsy Centre, Dianalund, Denmark; ^3^Department of Neuropediatrics, Children’s Hospital, University Medical Center Schleswig-Holstein, University of Kiel, Kiel, Germany; ^4^Institute of Clinical Molecular Biology, Christian-Albrechts-University of Kiel, Kiel, Germany; ^5^Department of Medical Genetics, Institute of Mother and Child, Warsaw, Poland; ^6^Division of Medical Genetics, Massachusetts General Hospital, Boston, MA, United States; ^7^The Saxon Epilepsy Center Kleinwachau, Radeberg, Germany; ^8^Department of General Pediatrics, Division of Child Neurology and Inherited Metabolic Diseases, Center for Pediatrics and Adolescent Medicine, University Hospital Heidelberg, Heidelberg, Germany; ^9^GeneDx, Gaithersburg, MD, United States; ^10^Research Centre for Medical Genetics, Moscow, Russia; ^11^Veltischev Research and Clinical Institute for Pediatrics of the Pirogov Russian National Research Medical University, Moscow, Russia; ^12^Department of Dermatology, The Nicolas Copernicus State Hospital, Koszalin, Poland; ^13^Department of Pediatrics, Endocrinology, Diabetology, Metabolic Diseases and Cardiology of the Developmental Age, Pomeranian Medical University, Szczecin, Poland; ^14^Department of Neurology, Keck School of Medicine, University of Southern California, Los Angeles, CA, United States; ^15^Department of Clinical Genomics, Laboratory of Medicine and Pathology, Center for Individualized Medicine, Mayo Clinic, Rochester, MN, United States; ^16^Department of Clinical Medicine, Faculty of Medical and Health Sciences, University of Copenhagen, Copenhagen, Denmark

**Keywords:** myoclonic-atonic seizures, generalized seizures, disease severity, developmental delay, glycosylphosphatidylinositol biosynthesis defects, inherited glycosylphosphatidylinositol-anchored protein (GPI-AP) deficiency

## Abstract

The two aims of this study were (i) to describe and expand the phenotypic spectrum of *PIGT* deficiency in affected individuals harboring the c.1582G>A; p.Val528Met or the c.1580A > G; p.Asn527Ser variant in either homozygous or compound heterozygous state, and (ii) to identify potential genotype-phenotype correlations and any differences in disease severity among individuals with and without the *PIGT* variants. The existing literature was searched to identify individuals with and without the two variants. A detailed phenotypic assessment was performed of 25 individuals (both novel and previously published) with the two *PIGT* variants. We compared severity of disease between individuals with and without these *PIGT* variants. Twenty-four individuals carried the *PIGT* variant Val528Met in either homozygous or compound heterozygous state, and one individual displayed the Asn527Ser variant in a compound heterozygous state. Disease severity in the individual with the Asn527Ser variant was compatible with that in the individuals harboring the Val528Met variant. While individuals without the Asn527Ser or Val528Met variant had focal epilepsy, profound developmental delay (DD), and risk of premature death, those with either of the two variants had moderate to severe DD and later onset of epilepsy with both focal and generalized seizures. Individuals homozygous for the Val528Met variant generally became seizure-free on monotherapy with antiepileptic drugs, compared to other *PIGT* individuals who were pharmaco-resistant. Two patients were diagnosed with myoclonic-atonic seizures, and a single patient was diagnosed with eyelid myoclonia. Our comprehensive analysis of this large cohort of previously published and novel individuals with *PIGT* variants broadens the phenotypical spectrum and shows that both Asn527Ser and Val528Met are associated with a milder phenotype and less severe outcome. Our data show that *PIGT* is a new candidate gene for myoclonic atonic epilepsy. Our genotype-phenotype correlation will be useful for future genetic counseling. Natural history studies of this mild spectrum of *PIGT-*related disorder may shed light on hitherto unknown aspects of this rare disorder.

## Introduction

Glycosylphosphatidylinositol (GPI) is a glycolipid that is synthetized and transferred to proteins in the membrane of the endoplasmic reticulum (Fujita and Kinoshita, 2012). The biogenesis of GPI-anchored proteins (GPI-APs) is a conserved post-translational mechanism in eukaryotes and is important for the attachment of these proteins to the cell membrane and for protein sorting, trafficking, and dynamics (Fujita and Kinoshita, 2012; Kinoshita and Fujita, 2016; Bayat et al., 2020b). GPI synthesis and GPI-AP modification are mediated by at least 31 genes, and pathogenic variants in 22 of these genes have so far been associated with human disease (Bellai-Dussault et al., 2019; Bayat et al., 2020b).

The *PIGT* gene encodes the phosphatidylinositol glycan class T protein that is part of the subunit of the heteropentameric GPI transamidase complex with *PIGK, PIGS, PIGT, PIGU*, and *PGAA1* that facilitates the attachment of GPI anchors to proteins (Ohishi et al., 2001, 2003).

Pathogenic germline missense and truncating variants in *PIGT* have been associated with a condition named “multiple congenital anomalies – hypotonia - seizures syndrome 3 (MCAHS3)” (OMIM 615398). Affected individuals share features such as epileptic seizures, severe to profound developmental delay (DD), intellectual disability (ID), and multiple congenital malformations of internal organs. Most individuals are non-verbal, non-ambulatory, and suffer from intractable epilepsy (Bayat et al., 2019). Generalized myoclonic and focal motor seizures with impaired awareness associated with various type of EEG epileptic abnormalities (focal sharp-slow wave, focal sharp spike/polyspikes wave, and generalized polyspikes-wave complexes) have been observed (Jezela-Stanek et al., 2020). While focal seizures with or without bilateral spreading have been described on several occasions (Bayat et al., 2019; Jezela-Stanek et al., 2020), reports of generalized seizures remain limited. Primary generalized seizures have so far only been described in a small number of individuals with a compound heterozygous variant at position Val528Met (Jezela-Stanek et al., 2020).

Individuals that are homozygous or compound heterozygous for the missense variant c.1582G > A; p.Val528Met have been reported to have a less severe phenotype (Pagnamenta et al., 2017; Bayat et al., 2019; Jezela-Stanek et al., 2020). This is currently the only variant reported to result in a milder phenotype. Amongst the 37 individuals described with *PIGT* deficiency (Kvarnung et al., 2013; Nakashima et al., 2014; Lam et al., 2015; Skauli et al., 2016; Knaus et al., 2018; Kohashi et al., 2018; Yang et al., 2018; Bayat et al., 2019; Mason et al., 2019; Jezela-Stanek et al., 2020; Jiao et al., 2020), only 13 individuals have been reported to harbor this variant in a homozygous or compound heterozygous state (Pagnamenta et al., 2017; Bayat et al., 2019; Jezela-Stanek et al., 2020). Our knowledge remains limited about the extent of DD and ID associated with this variant and about the natural history in adolescence and adulthood. Furthermore, a detailed comparison of symptoms in affected individuals with and without the p.Val528Met variant has yet to be done. More individuals are needed to better delineate the genotype-phenotype correlation and to investigate if other pathogenic variants can also be associated with a milder phenotype.

Here, we describe 13 novel individuals with a *PIGT* deficiency and 12 previously reported individuals (Bayat et al., 2019; Jezela-Stanek et al., 2020). We also present follow-up data on two of the 12 previously published individuals [P4 and P5 from Bayat et al. (2019)]. Of the 25 individuals, 24 harbored the Val528Met variant in either homozygous or compound heterozygous state, while one individual had a previously unreported c.1580A > G; p.Asn527Ser variant. We present a detailed phenotyping of the affected individuals and expand the known epilepsy semiology to include myoclonic-atonic seizures. We also compare the findings with those seen in previously published affected individuals without the two variants.

## Materials and Methods

### Individual Analysis

Subjects were identified and recruited either through their treating clinicians, self-referral, or GeneMatcher (Sobreira et al., 2015). Basic medical information on birth parameters, epilepsy, developmental history, and physical examinations was collected from healthcare providers and/or families of affected individuals. Molecular testing results from exome sequencing were submitted by healthcare providers. In eight cases, individual videos provided by caregivers were also used to evaluate motor skills. All the videos submitted and evaluated were used with the written consent of guardians. Videos were evaluated by authors AB and GR.

The clinical findings of this cohort were compared to previously published individuals with *PIGT* deficiency who did not harbor either of the two variants. As the access to clinical data was limited in a substantial number of published individuals, we chose only to compare data on seizures and neurological development.

### Standard Protocol Approvals, Registrations, and Individual Consent

The study was conducted in agreement with the Declaration of Helsinki and approved by the local ethics committee of the referring center. As all individials were either minors or had cognitive impairment, their parents or legal guardians gave informed consent. The clinical information was collected through interviews with the families and/or from hospital records of the individuals and their family members. Videos were collected from either caregivers or healthcare providers.

### Literature Search

We searched MEDLINE (PubMed) with the keywords epilepsy, GPI, GPI-AP, or GPI-anchored protein in combination with *PIGT* or phosphatidylinositol glycan class T protein (last PubMed search: January 2021). Any relevant references in the assessed articles, which were not found in the MEDLINE search, were further investigated. Only articles written in English and published after 1980 were included to ensure optimal data collection. Only cases with a confirmed molecular diagnosis were included.

### Data Availability

Anonymized data, including data not published in this article, will be made available on request from any qualified investigator.

### Genetic Identification and Analysis

All affected individuals were investigated by gene panels or exome sequencing ordered by healthcare providers. Variants were classified based on the 2015 joint guidelines from the American College of Medical Genetics and Genomics and the Association for Molecular Pathology (Richards et al., 2015). The protein-coding transcript used was NM_015937.6. We predicted the functional alteration of novel variants using polymorphism phenotyping-2 (PolyPhen-2) (Adzhubei et al., 2010) and SIFT (Sorting intolerant from tolerant) (Farheen et al., 2017), CADD^1^, and mutationTaster^2^.

## Results

### Individuals and Families

We collected 13 living, novel *PIGT*-deficient individuals (8 males and 5 females) harboring either the Val528Met or the Asn527Ser variant. In addition, we present follow-up data for two previously published individuals [P4 and P5 published by Bayat et al. (2019)] 2 years after the initial publication. Fourteen individuals carried the Val528Met variant in either homozygous (*n* = 6) or compound heterozygous (*n* = 8) state, while a single individual had the Asn527Ser variant in a compound heterozygous state.

From the available literature, we identified ten previously reported individuals with the Val528Met variant [Decipher ID 258094 by Pagnamenta et al. (2017), P3 and P10 by Bayat et al. (2019), and P1-P7 by Jezela-Stanek et al. (2020)]. Table 1 provides an overview of the clinical and genetic findings.

**TABLE 1A:** Clinical and genetic findings in 25 novel and previously published individuals with *PIGT* deficiency harboring the p.Asn527Ser or p.Val528Met variant in either homozygous or compound heterozygous state.

Families	Family 1	Family 2	Family 3	Family 4	Family 5	Family 6	Family 7	Family 7	Family 8	Family 9	Family 10	Family 10	Family 10

	Patient 1	Patient 2	Patient 3	Patient 4	Patient 5	Patient 6	Patient 7	Patient 8	Patient 9	Patient 10	Patient 11	Patient 12	Patient 13
cDNA change (NM_015937.6)	c.918dupC; c.1580A > G	c.769+2T > A; c.1582G > A	c.988C > T; c.1582G > A	c.988C > T; c.1582G > A	c.1127A > C; c.1582G > A	c.1342C > T; c.1582G > A	c.1519C > T; c.1582G > A	c.1519C > T; c.1582G > A	c.1520G > A; c.1582G > A	c.1582G > A; c.1582G > A	c.1582G > A; c.1582G > A	c.1582G > A; c.1582G > A	c.1582G > A; c.1582G > A

Amino acid change (NP_057021.2)	p.Val307Arg fsTer13; p.Asn527Ser	Splice site; p.Val528Met	p.Arg330Ter; p.Val528Met	p.Arg330Ter; p.Val528Met	p.His376Pro; p.Val528Met	p.Arg448Trp; p.Val528Met	p.Arg507Trp; p.Val528Met	p.Arg507Trp; p.Val528Met	p.Ala507Gln; p.Val528Met	p.Val528Met; p.Val528Met	p.Val528Met; p.Val528Met	p.Val528Met; p.Val528Met	p.Val528Met; p.Val528Met

Clinical significance	PATH; PATH	PATH: PATH	PATH; PATH	PATH; PATH	PATH; PATH	PATH; PATH	PATH; PATH	PATH; PATH	PATH; PATH	PATH; PATH	PATH; PATH	PATH; PATH	PATH; PATH

Heritage	Caucasian, African, Native American	Caucasian	Caucasian	Asian	Caucasian/African	Caucasian	Russian	Russian	Polish	Russian	Caucasian	Caucasian	Caucasian

Age at inclusion	2 years	16 years	28 years	6 years	2.6 years	6 years	8 years	22 years	3 years	6.5 years	11 years	4 years	2 years

Gender	Male	Male	Female	Female	Female	Female	Male	Female	Male	Female	Female	Female	Female

Seizures	Yes	Yes	Yes	Yes	No	Yes	Yes	Yes	No	Yes	Yes	Yes	No

Epilepsy diagnosis	Yes	Yes	Yes	No (febrile seizures only)	No	Yes	Yes	Yes	No	Yes	Yes	Yes	No

Age at seizure onset	Unknown	2 years 9 months	5 years 5 months	3 years	Not relevant	5 months	5 months	18 months	Not relevant	12 months	11 months	7 months	Not relevant

Non-epileptic myoclonic jerks	Unknown	Unknown	Yes (intractable to treatment)	No	No	No	Yes	No	No	No	Unknown	Unknown	Unknown

Seizure types during disease course	Single event with bilateral tonic–clonic seizure	Focal MS, focal hypomotor seizures with impaired awareness, FBTCS	FBTCS	Fever-induced bilateral tonic– clonic seizures	Not relevant	FBTCS	MAE, eyelid- myoclonia	MAE	Not relevant	Focal hypomotor seizures with impaired awareness, FBTCS	FBTCS	FBTCS	Unknown

Febrile seizures	No	No	Yes	Yes	Not relevant	Yes	No	No	Not relevant	Yes	No	No	No

Status epilepticus	No	No	Yes	No	Not relevant	No	No	No	Not relevant	N	No	No	Unknown

Current AED	None	None	LMT, VPA, CBD	None	Not relevant	LMT, VPA	None	None	Not relevant	LEV	VPA	VPA	None

Lifetime AED	None	LMT, TPM, LEV, VPA	LMT, VPA, CBD, CBZ, PHT, PMD, CLB	None	Not relevant	LMT, VPA	CBZ	CBZ and VPA	Not relevant	LEV	VPA	VPA	None

Overall antiepileptic drug response^∗^	Not relevant	Very good	Partially good (free of epileptic seizures but not the non- epileptic myoclonic jerks)	Never used	Not relevant	Very good	Very good	Very good	Not relevant	Very good	Very good	Very good	Not relevant

Age at sitting	Unknown butcan sit unaided	Unknown butcan sit unaided	19 months	2 years	22 months	2.5 years	2.5 years	2 years	13 months	4 years	3 years	2 years	1.5 years

Age at walking	Unknown but can walk unaided	Unknown but can walk unaided	8 years (assisted)	3 years (assisted)	Unable	4 years	Unable	4 years	27 months (assisted, short distances)	3 years	5 years	Unable	22 months (assisted)

Useful use of hands	Yes	Yes	Yes	Yes	Yes	Yes	Yes	Yes	Yes	Unknown	Yes	Yes	Yes

Age at first words	Unknown but can talk	Unknown but can talk	9 months	3 years	14 months (mama)	16 months	Only simple sounds	Only simple sounds	15 months	4 years	2.5 years	3 years	18 months

Present verbal ability	Understands simple sentences. Uses simple words	Understands simple sentences. Has a vocabulary of 100 single words. Dysarthric speech	Understands simple sentences. Replies with single words or short sentences. Dysarthric speech	Understands simple sentences. Has a vocabulary of 100 single words and in addition uses sign language	Nonverbal besides “mama”	Understands simple sentences. Uses 10 single words and has started to combine two words	Non-verbal	Non-verbal	Understands simple sentences. Single words, dysarthria. Uses sign language	Single words	Understands most sentences. Has a vocabulary of > 100 single words and can use short sentences. Dysarthric speech	Understands simple sentences. Uses 10 single words and has started to combine two words	Understands simple sentences. Uses a few words

Developmental delay	Moderate	Moderate	Moderate	Moderate– severe	Moderate– severe	Moderate	Severe	Severe	Moderate– severe	Moderate– severe	Moderate	Moderate– severe	Moderate– severe

Autism	No	No	No	No	No	No	No	No	No	No	No	No	No

Brain MRI results	Not performed	12 months: delayed myelination; 8 and 14 years: pronounced cerebellar atrophy	22 months: cerebellar atrophy; 11, 14, and 16 years: thinning of CC, progressive atrophy of cerebellum, pons, and mesencephalon; 25 years: hippocampal sclerosis	12 months: delayed, myelination	Not performed	Not performed	10 months: delayed myelination and enlargement of the subarachnoid spaces	15 years: cerebellar atrophy and thinning of CC	10 months: delayed myelination; 31 months: cortical and cerebellar atrophy	4 years: delayed myelination; 5 years: unremarkable	Not performed	Not performed	Not performed

*Overall antiepileptic drug response (very good = seizure-free; partially good = partially seizure-free on treatment; and poor = intractable seizures despite treatment).

**TABLE 1B:** Clinical and genetic findings in novel and previously published individuals with *PIGT* deficiency harboring the p.Asn527Ser or p.Val528Met variant in either homozygous or compound heterozygous state.

Families	Family 11	Family 11	Family 12	Family 13	Family 14	Family 15	Family 16	Family 17	Family 18	Family 19	Family 20	Family 21

	Patient 14	Patient 15	P1 (Jezela-Stanek et al., 2020)	P2 (Jezela-Stanek et al., 2020)	P3 (Jezela-Stanek et al., 2020)	P4 (Jezela-Stanek et al., 2020)	P5 (Jezela-Stanek et al., 2020)	P6 (Jezela-Stanek et al., 2020)	P7 (Jezela-Stanek et al., 2020)	P3 (Bayat et al., 2019)	P10 (Bayat et al., 2019)	Decipher ID 258094 (Pagnamenta et al., 2017)
cDNA change (NM_015937.6)	c.1582G > A; c.1582G > A	c.1582G > A; c.1582G > A	c.1582G > A; c.1582G > A	c.1730dupC; c.1582G > A	c.1520G > A; c.1582G > A	c.494-2A > G; c.1582G > A	c.1582G > A; c.1582G > A	c.1096G > A; c.1582G > A	c.494-2A > G; c.1582G > A	c.494-2A, c.1582G > A;	c.1724_1725insC; c.1582G > A	c.1730dupC; c.1582G > A

Amino acid change (NP_057021.2)	p.Val528Met; p.Val528Met	p.Val528Met; p.Val528Met	p.Val528Met; p.Val528Met	p.Leu578fsTer 35; p.Val528Met	p.Ala507Gly; p.Val528Met	Splice site; p.Val528Met	p.Val528Met; p.Val528Met	p.Gly366TArg; p.Val528Met	Splice site; p.Val528Met	Splice site; p.Val528Met	p.Leu578fsTer35; p.Val528Met	p.Leu578fsTer35; Val528Met

Clinical significance	PATH; PATH	PATH; PATH	PATH; PATH	PATH; PATH	PATH; PATH	PATH; PATH	PATH; PATH	PATH; PATH	PATH; PATH	PATH; PATH	VUS;PATH	PATH; PATH

Heritage	Caucasian	Caucasian	Polish	Polish	Polish	Polish	Polish	Polish	Polish	Polish	Polish	Polish

Age at inclusion	2 years	7.5 years	Born 2019	Born 2017	Born 2017	Born 2016	Born 2011	Born 2013	Born 2003	5 years	4 years	Unknown

Gender	Female	Female	Female	Male	Male	Male	Female	Male	Female	Female	Female	Female

Seizures	Yes	Yes	Yes	Yes	Yes	Yes	Yes	Yes	Yes	Yes	Yes	Yes

Epilepsy diagnosis	Yes	Yes	No	No	Yes	Yes	Yes	No	No	Yes	Yes	Yes

Age at seizure onset	6 months	20 months	6 months	12 months	6 months	9 months	6 months	11 months	12 months	12 months	18 months	12 months

Non-epileptic myoclonic jerks	Yes	No	Unknown	Unknown	Unknown	Unknown	Unknown	Unknown	Unknown	Unknown	Unknown	Unknown

Seizure types during disease course	Atypical absences (generalized)	Atypical absences	Fever-induced tonic–clonic seizures of unknown onset	Fever-induced focal seizures with impaired awareness	Focal hypomotor seizures with impaired awareness, FBTCS	Absences, myoclonic jerks, and bilateral tonic–clonic seizures	Focal hypomotor seizures with impaired awareness, FBTCS, and generalized myoclonic jerks	Focal hypomotor seizures with impaired awareness	Focal hypomotor seizures with impaired awareness	Fever-induced tonic–clonic seizures of unknown onset	Bilateral tonic–clonic seizures of unknown onset	Bilateral tonic–clonic seizures of unknown onset

Febrile seizures	No	Yes	Yes (only)	Yes (only)	Yes	Yes	Yes	Yes (only)	Yes	Unknown	Unknown	Unknown

Status epilepticus	No	No	Unknown	Unknown	Unknown	Unknown	Unknown	Unknown	Unknown	Unknown	Unknown	Unknown

Current AED	LEV	LEV	None	Unknown	Unknown	Unknown	Unknown	Unknown	Unknown	Unknown	Unknown	Unknown

Lifetime AED	LEV	LEV	None	Unknown	Unknown	Unknown	Unknown	Unknown	Unknown	Unknown	Unknown	Unknown

Overall antiepileptic drug response^∗^	Very good	Very good	Not relevant	Very good	Very good	Poor (intractable myoclonic jerks)	Good	Very good	Very good	Good	Good	Good

Age at sitting	1 year (with support)	3 years	Unable	25 months	18 months	30 months	2.5 years	2 years	2.5 years	Unknown	Unknown	Unknown

Age at walking	Unable	6 years (a few steps)	Unable	Unknown but can walk assisted	Unable	Unable	Unable	Unable	6 years (unassisted but short distances)	Unknown	Unknown	Unknown but can walk

Useful use of hands	Unknown	Unknown	Unknown	Unknown	Unknown	Unknown	Unknown	Unknown	Unknown	Unknown	Unknown	Unknown

Age at first words	Non-verbal	Non-verbal	Unknown	Unknown	Unknown	23 months	3.5 years	4.5 years	5 years	Unknown	Unknown	Unknown

Present verbal ability	Unknown	Understands simple sentences. Uses a communication device with eye movement control for words	Unknown	Unknown	Single words, dysarthria	Single words	Communicates through simple sentences, dysarthria	Single words	Single words, dysarthria	Unknown	Unknown	Unknown

Developmental delay	Severe	Severe	Unable to classify	Unable to classify	Unable to classify	Unable to classify	Unable to classify	Unable to classify	Unable to classify	Unable to classify	Unable to classify	Unable to classify

Autism	Unknown but has limited eye contact	No	Unknown	Unknown	Unknown	Unknown	Unknown	Unknown	Unknown	Unknown	Unknown	Unknown

Brain MRI results	Not performed	2 years: cerebellar atrophy	6 months: delayed myelination	10 months: delayed myelination, slight enlargement of pericerebral spaces	12 months: decreased white matter volume, enlargement of pericerebral spaces	12 months: decreased cerebral white matter volume including CC; 16 months: cortical–subcortical atrophy	8 months: delayed myelination and slight enlargement of pericerebral spaces; 3 years: cortical–subcortical and cerebellar vermis atrophy	Three times over 1 year: progressive cortical–subcortical atrophy, cerebellar vermis atrophy	12 months: normal; 11 and 16 years: progressive cerebral and cerebellar atrophy	Unknown age: cerebellar atrophy	4 years: progressive isolated cerebellar atrophy	Progressive isolated cerebellar atrophy

*Overall antiepileptic drug response (very good = seizure-free; partially good = partially seizure- free on treatment; and poor = intractable seizures despite treatment). Abbreviations: AED, antiepileptic drug; CBD, cannabidiol; CBZ, carbamazepine; CC, corpus callosum; EEG, electroencephalogram; FBTCS, focal to bilateral tonic–clonic seizure; LEV, levetiracetam; LMT, lamotrigine; MAE, myoclonic atonic epilepsy; MRI, magnetic resonance imaging; MS, myoclonic seizure; PATH, pathogenic; PHT, phenytoin; PIGT, phosphatidylinositol glycan class T protein; PMD, primidone; VPA, valproate; VUS, variant on unknown significance; TPM, topiramate.

Part 1 of Table 2 compares the clinical findings in the 25 individuals with the Val528Met or the Asn527Ser variant with those of 24 published individuals without these two *PIGT* variants (Kvarnung et al., 2013; Nakashima et al., 2014; Lam et al., 2015; Skauli et al., 2016; Knaus et al., 2018; Kohashi et al., 2018; Yang et al., 2018; Bayat et al., 2019; Mason et al., 2019; Jiao et al., 2020).

**TABLE 2:** Brief clinical overview of all published individuals with *PIGT* deficiency.

	**Part 1. Grouping of individuals (*n* = 49) based on whether they have the c.1582G>A; p.Val528Met or the c.1580A>G; p.Asn527Ser variant**		**Part 2. Grouping of individuals (*n* = 25), with at least one copy of the c.1582G>A; p.Val528Met or the c.1580A>G; p.Asn527Ser variant, based on annotation of the disease causing variant on the other allel**		
		
	Individuals with the p.Val528Met or the p.Asn527Ser variant	Individuals without the p.Val528Met or the p.Asn527Ser variant	Individuals homozygous for the p.Val528Met variant	Individuals compound heterozygous for a missense variant in addition to the p.Val528Met or p.Asn527Ser variant	Individuals compound heterozygous for a truncating variant in addition to the p.Val528Met or p.Asn527Ser variant
Total number	25	24	8	7	10

Gender ratio (M/F)	9/16	14/10	0/8	4/3	4/6

Epileptic seizures	22/25 (88%)	24/24 (100%)	7/8 (87%)	5/7 (71%)	10/10 (100%)

Only febrile seizures	5/25 (20%)	1/24 (4%)	1/7 (15%)	1/5 (20%)	3/10 (30%)

Epilepsy diagnosis	17/22 (77%)	23/24 (96%)	6/7 (85%)	4/5 (80%)	8/10 (80%)

Average onset of seizures	16 months	6 months	9 months	9 months	23 months

Overall seizure outcome	Good (24/25, 96% seizure-free)	Bad (23/24, 96% intractable)	Good	Good	Good

Non-epileptic myoclonic jerks	2/25	Unknown	0/8	1/7	1/10

Degree of developmental delay	Moderate (5/25), moderate-severe (6/25), severe (4/25), unable to ascertain (10/25)	Profound (24/24, 100%)	Moderate (1/8), moderate-severe (3/8), severe (2/8), unable to ascertain (2/8)	Moderate (1/7), moderate-severe (2/7), severe (2/7), unable to ascertain (2/7)	Moderate (3/10), moderate-severe (1/10), severe (0/10), unable to ascertain (6/10)

Congenital hypotonia	24/25 (96%)	23/24 (96%)	7/8	7/7	10/10

Premature mortality	0/25 (0%)	3/23 (13%)	0/8	0/7	0/10

Landscape of disease causing variants	Patients with compound missense/truncating (*n* = 10), biallelic missense (*n* = 15), or biallelic truncating (*n* = 0) variants.	Patients with compound missense/truncating (*n* = 6), biallelic missense (*n* = 18), or biallelic truncating (*n* = 0) variants.			

### Phenotypic Analysis

In total, we evaluated the phenotypical features of 25 living individuals, including 15 novel and 10 previously published individuals (Bayat et al., 2020b; Jezela-Stanek et al., 2020).

#### Perinatal History

Few complications occurred during pregnancy. The 20-week ultrasound of one individual (P13) showed cardiac septal defects and decreased fetal movements while the other 24 patients did not exhibit any malformations on prenatal ultrasonography. The gestation length was at least 36 weeks (36–41 weeks) in all cases and was at term for the vast majority of deliveries. All children had either normal or increased birth parameters. The mean weight and length at birth were 3.9 kg (3.2–4.5 kg) and 54 cm (49–60 cm).

There were no reported stillbirth cases and no terminated pregnancies. Data on prenatal history are not shown.

#### Ophthalmological and Otological Features

Data on ophthalmological features were available for 22/25 (88%) individuals. Strabismus was diagnosed in 15/25 (60%), refractive error in 10/25 (40%), and cortical visual impairment (CVI) was evident in 7/25 (28%).

Individual P1 with the Asn527Ser variant had neither CVI, strabismus, nor a refractive error. Instead, he was diagnosed with features such as microphthalmia, posterior synechiae of the anterior chamber, partial angle closure, tight falciform retinal folds, peripheral avascular retina, retinal hemorrhage, and extensive vitreous membranes. Although one individual with the Asn527Ser variant exhibited ophthalmological features not previously described in *PIGT* deficiency, her overall phenotype was compatible with those found in affected individuals with the Val528Met variant. Exome sequencing found no additional explanation for the ophthalmological features. Functional testing by flow cytometry to determine the surface levels of GPI-APs (Knaus et al., 2018) was unfortunately unavailable for this subject. Such testing is needed in future patients with Asn527Ser variants to prove pathogenicity and to perform deep-phenotyping of the clinical features including any anomalies of the eyes.

Otological features have only rarely been addressed previously. We identified 2/25 (8%) individuals who had suffered from hearing disorders; one presented with a sensorineural deficit (P1), and the other with an unspecified impairment (P4) (no further data were available).

#### Cardiovascular and Gastrointestinal Features

While congenital heart defects have been associated with other pathogenic variants of the *PIGT* gene (Bayat et al., 2019), we cannot report any heart anomalies in individuals with Asn527Ser and Val528Met. Echocardiography was available in 10/25 individuals and was normal in all probands.

Data on gastrointestinal disturbances were available in 16/25 individuals. None of the individuals required a gastrostomy tube. Three individuals experienced feeding difficulties during the first 2 months of life, and four experienced gastroesophageal reflux and recurrent constipation.

#### Neurological and Behavioral Phenotype

Epileptic seizures were reported in 22/25 individuals (88%) [12 individuals from our cohort, Decipher ID 258094 (Pagnamenta et al., 2017) P1-P7 (Jezela-Stanek et al., 2020), P3 and P10 (Bayat et al., 2019)], but only 18 individuals fulfilled the diagnostic criteria for having epilepsy [11 individuals from our cohort, Decipher ID 258094 (Pagnamenta et al., 2017), P3-5 and P7 (Jezela-Stanek et al., 2020), P3 and P10 (Bayat et al., 2019)]. In 1/18 individuals (P1) only a single afebrile seizure was reported, but an epilepsy diagnosis was given due to the additional finding of interictal epileptic discharges on electroencephalogram (EEG). Four individuals [P1, P2, and P6 (Jezela-Stanek et al., 2020), and our P4] had febrile seizures as the only seizure type and did not reach an epilepsy diagnosis. Finally, febrile seizures were present in 13/25 (52%) individuals.

Age at seizure onset was available in all but one individual. The average time of onset was around 16 months of age (from 5 months to 5.5 years). Twelve individuals experienced their first epileptic seizure during the 5th to 12th month of life while the remaining children had an onset around the 2nd to 5th year of life (Table 1).

Seizure types were either focal (12/25) or primary generalized (9/25). The predominant seizure type was focal to bilateral tonic-clonic seizures, followed by primary generalized bilateral tonic-clonic seizures. Other generalized seizure types included atypical absences (3/25), myoclonic seizures (2/25), myoclonic-atonic seizures (2/25), and eyelid myoclonia (1/25). Only one individual experienced status epilepticus (P3).

Antiepileptic drugs (AEDs) were prescribed in 18/25 individuals. Pagnamenta et al. (2017) provided no data on AED treatment. Several of the individuals described by Jezela-Stanek et al. (2020) were taking AEDs despite only having febrile seizures and thereby not fulfilling the criteria for epilepsy; these authors provided no data on the type of AEDs used. Thus, a detailed description of types of AEDs prescribed in the 18 individuals was only possible for 10 individuals, all of whom fulfilled an epilepsy diagnosis. Six of these ten individuals became seizure-free on monotherapy with either valproate (VPA) or levetiracetam. Three of the ten individuals (P2, P3, and P6) became free of epileptic seizures on a combination of VPA and lamotrigine, but individual P3 had persistent non-epileptic myoclonic jerks. One individual (P7) became seizure-free on monotherapy with carbamazepine. Three of the ten individuals had been tapered off from their AED(s) and have been seizure-free for several years.

Comparison of symptoms seen in individuals homozygous for the Val528Met variant to those found in individuals compound heterozygous for Asn527Ser or Val528Met, revealed no convincing difference regarding onset of seizures, seizure types, and cognitive and developmental outcome. Although focal and primary generalized seizures were present in both subgroups, we noticed that atypical absence seizures were only present among the homozygous individuals and that homozygous individuals became seizure-free on monotherapy with AED. Compound heterozygous individuals typically needed a combination of AEDs to achieve seizure freedom. We also explored if it was possible to draw further genotype-phenotype conclusions within the group of 25 individuals (carrying the Asn527Ser or Val528Met variant) based on the annotation of the disease causing variant found on the other allel. In part 2 of Table 2 the 25 individuals were divided in three groups: (i) individuals homozygous for the p.Val528Met variant, (ii) individuals compound heterozygous for a missense variant in addition to the p.Val528Met or p.Asn527Ser variant, and (iii) individuals compound heterozygous for a truncating variant in addition to the p.Val528Met or p.Asn527Ser variant. While the majority of individuals in the two first groups experienced seizures this was the case for all individuals in the third group. A small subset of individuals in all groups only experienced fever induced seizures and thus did not reach a formal diagnosis of epilepsy (Table 2, part 2). In all three groups 80–85% of individuals with seizures reached a formal diagnosis of epilepsy. In the first two groups the age of onset of seizures was around 9 months of life while seizures started around 2 years of age in the third group. Individuals carrying a disease causing missense variant on both allels were compatible in regard of the degree of DD. The overall seizure outcome across all groups was good as all but one individual became seizure free after treatment initiation of AEDs (Table 2).

**FIGURE 1:**
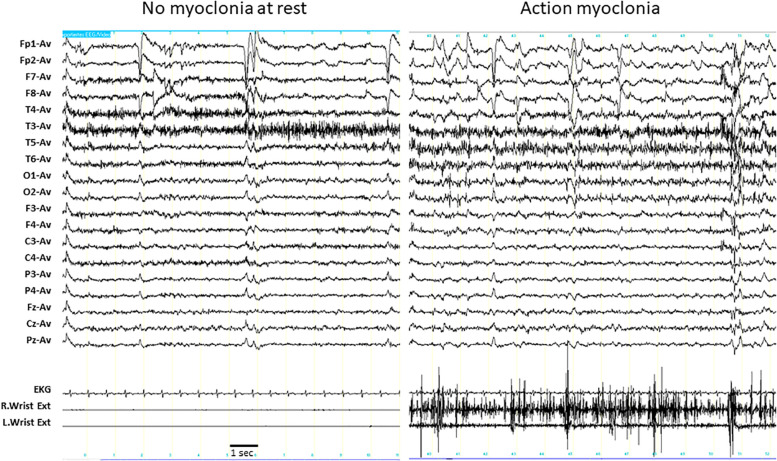
EEG polygraphic recording in individual P3. **Left panel:** the individual is at rest. EEG does not show any epileptic abnormalities, and the myoclonic EMG activity is detectable in both wrist extensors. **Right panel:** the individual is maintaining a posture (outstretching her arms). The muscular contraction in both wrist extensors are fragmented by irregular, high amplitude, myoclonic potentials that are intermixed with brief silent periods, reproducing the EMG pattern of action myoclonus. No epileptic discharges associated with the myoclonic potentials were detectable in the EEG. Wrist Ext, wrist extensor; R, right; L, left.

Massive action myoclonia involving all four limbs and the trunk were observed in individual P3 without any clinical and polygraphic evidence of myoclonus at rest (Supplementary Video 1). This phenomenon started around the age of 8 years and became progressively worse and disabling during the course of the disease. No epileptic abnormalities were associated with the myoclonic phenomena on EEG (Figure 1).

The age range of the 25 individuals spanned from 27 months to 29 years. Degree of DD was as follows: moderate (5/25), moderate-severe (6/25), and severe (4/25). In 10 individuals [Decipher ID 258094 (Pagnamenta et al., 2017), P1–7 (Jezela-Stanek et al., 2020), and P3 and P10 (Bayat et al., 2019)], we were unable to obtain enough clinical data to assess the degree of DD, notably none of the individuals had profound DD (Table 2, part 1). Of the 25 individuals, 13 eventually learned to walk: 11 could walk short distances with walking aids or support from others, and two individuals learned to walk independently. The average age of independent walking was between the 3rd and 6th year of life. We were able to collect data on manual dexterity in 13 individuals. All individuals had purposeful use of hands and could eat autonomously using cutlery, play with bricks or puzzles, or use sign language. Information on verbal capabilities was obtained from 19 individuals. All 19 individuals comprehended simple sentences and 15/19 replied using single words. Two individuals, both homozygous for the Val528Met variant, could communicate with very simple sentences: one individual (P11) was even able to educate her younger affected sibling (P12) (Supplementary Video 2). Two individuals (currently 2 and 7.5 years of age) remained non-verbal. None of the patients were diagnosed with an autism spectrum disorder.

#### Electroencephalogram

EEG findings were available in five individuals with epilepsy (P2, P3, P7, P8, and P10). A poorly organized background activity was reported in two individuals, with burst of theta activity intermixed. Generalized epileptic discharges with frontal predominance were reported in two individuals (P8 and P10) at the age of 14 and 3 years, respectively. Occasional focal epileptic discharges were observed in individual P2 at the age of 3 years but were not detected after the age of 14 years, whereas isolated focal spikes were observed at the age of 10 months in individual P7. Individual P3, who exhibited action myoclonus, did not exhibit interictal epileptic abnormalities on routine EEG.

#### Brain Imaging

At least one brain MRI scan was performed in 18/25 individuals (Table 1), and 12 of those underwent repeated MRI scans. In six individuals, the first cerebral MRI was performed during the first year of life (in all cases between the 6th and 12th month of life). Delayed myelination was noted in all six individuals, while enlargement of the subarachnoid spaces was detected in three of them. There were no follow-up data on the delayed myelination described in our individuals P4 and P7, but the myelination was age-appropriate in the remaining four individuals.

MRI was performed in twelve individuals after the age of 1 year. Scans showed age-appropriate myelination associated with several developmental anomalies such as cerebellar atrophy (11/12) occasionally involving the brainstem (1/12), and abnormal corpus callosum (3/12). Additional MRI findings included prominent cortical and subcortical volume loss (7/12), enlargement of lateral and third ventricles (3/12), white matter immaturity (2/12), and a hippocampal sclerosis (1/12) (P3). In individual P10, the MRI detected delayed myelination at the age of 4 years, but the MRI scan a year later was unremarkable (Table 1).

We have previously shown that cerebral MRI findings of individuals with *PIGT* deficiency include delayed myelination, prominent cortical and subcortical volume loss with brainstem atrophy, atrophy of the cerebellum, and an abnormal corpus callosum (Bayat et al., 2019). This is compatible with the current results from our individuals with Asn527Ser and Val528Met. The natural history of the delayed myelination in other *PIGT* variants has to be delineated further, but the present study indicates that the myelination eventually becomes age-appropriate in individuals carrying Asn527Ser and Val528Met variants. Only one individual exhibited an unremarkable MRI at 5 years of age (P9). Only few *PIGT-*deficient individuals without those variants have been reported to have normal cerebral MRIs (Bayat et al., 2019). The examinations were done at a very early age, however, which might explain why no structural anomalies were found.

#### Molecular Results

From the complete dataset of the cohort of 25 individuals, 13 different variant sites emerged: seven missense variants, two splice site variants, and four truncating variants. The Val528Met variant has been reported in 29 out of 282.654 samples (0.0001026 allele frequency)^3^. In comparison, the Asn527Ser variant has only been reported once in 251.292 samples (0.000003979 allele frequency) and is predicted to be damaging by all four prediction tools (Supplementary Table 1) and likely to be damaging according to the ACMG guidelines (Richards et al., 2015). The clinical findings in the individual with the Asn527Ser was compatible with those harboring the Val528Met variant. While the previously published variant c.1724_1725insC; p.Leu578fsTer35 (Bayat et al., 2019) was classified as a variant of unknown significance, all remaining variants were predicted as either likely pathogenic or pathogenic (Supplementary Table 1).

## Discussion

In 2017, Pagnamenta et al. (2017) reported two individuals with *PIGT* deficiency: one was compound heterozygous for the variants Val528Met and Leu578^∗^35, while the second was homozygous for the Glu237Gln. Western blot analysis showed that the expression of the mutant protein appeared to be reduced only for the truncating variant (8). *PIGT*-knockout HEK293 cells were then transfected with wild-type or mutant *PIGT* cDNA. The largest fraction of CD59-rescued cells was observed with Val528Met, indicating highest residual functional activity (Pagnamenta et al., 2017). While the individual with the Glu237Gln was profoundly affected, the individual with the Val528Met and Leu578^∗^35 variants was significantly less affected. This individual walked unaided but with an ataxic gate, understood spoken language but exhibited dysarthric speech, and attended a mainstream school with one-to-one support. This was the first evidence suggesting that the Val528Met is associated with a less severe phenotype, and our data give further support to this claim. Bayat et al. (2019) described four individuals harboring the Val528Met variant in either homozygous or compound heterozygous state, and in 2020 an additional seven individuals were described (Jezela-Stanek et al., 2020). Our knowledge of the phenotypical spectrum associated with homozygous and compound heterozygous Val528Met variants remains limited. We also have little insight into the natural history of this rare disorder. This is a challenge when counseling families with newly diagnosed children with Val528Met-related *PIGT* deficiency.

We compared the group of *PIGT* individuals with the Asn527Ser or the Val528Met (in either homozygous or compound heterozygous state) to a group of *PIGT* individuals without such variants (Table 2). In both groups (Table 2, part 1), the majority of individuals carried biallelic missense variants while there were no cases with biallelic truncations as this would likely not be compatible with life. We also explored potential genotype-phenotype correlations within the group of individuals with the Asn527Ser or Val528Met variant and grouped individuals based on the annotation of the disease causing variant found on the other allel (Table 2, part 2). In the group of individuals that were compound heterozygous for a truncating variant in addition to the p.Val528Met or p.Asn527Ser variant all experienced epileptic seizures with an average onset around 2 years of age. We were unable to detect other genotype-phenotype correlations based on the present dataset (Table 2, part 2). Studies of rare diseases are often conducted through a multicenter approach, and researchers are dependent on data provided by caregivers and other health care providers. This gives an unavoidable data collection bias as patients are evaluated by diverse health care providers with different medical backgrounds and levels of experience. In the current study, we aimed to reduce this bias by collecting videos of individuals with the two variants. These videos of eight individuals were used to give a more uniform evaluation of motor, verbal social, and cognitive skills. We found a more favorable outcome in our individuals, with less severe global DD, a considerably later onset of epilepsy, and no premature mortality (Table 2). While no individuals with the Asn527Ser and Val528Met variants were diseased, 3/24 (13%) individuals without the variants in question died prematurely (Bayat et al., 2019). Although the cumulative mortality rate is lower compared to what has been described in other genes involved in the GPI anchor synthesis (Bayat et al., 2020a), this finding gives more strength to the claim that the two *PIGT* variants are associated with a milder phenotype and a better prognosis.

Epilepsy was not a constant feature in our cohort although when present, it was treatment-responsive. While all previously identified individuals with different pathogenic variants had epilepsy and a more profound global DD, the individuals with Asn527Ser or Val528Met ranged from moderate to severe global DD (Table 2, part 1). We could also show that a minority of individuals with the Val528Met variant experienced only febrile seizures or had never had epileptic seizures (Table 1). Individuals with the two variants generally became seizure-free on monotherapy with AEDs, compared with other *PIGT* individuals that were drug-refractory (Table 2).

The comparison of individuals with homozygous Val528Met variants and those carrying compound heterozygous variants Asn527Ser and Val528Met lead to the conclusion that homozygosity is associated with a better outcome. Our study gives evidence to the claim that Asn527Ser- and Val528Met-related *PIGT* deficiency is associated with a mild to moderate “developmental and epileptic encephalopathy (DEE)”, as compared to *PIGT*-related DEE caused by other pathogenic variants. A similar spectrum of symptoms ranging from mild to severe DEE is seen in the X-linked phosphatidylinositol glycan class A protein gene that is involved in one of the first steps of GPI anchor biosynthesis (Bayat et al., 2020b). As in other DEEs, co-existence of focal and generalized seizures in the same individual during a lifetime is a feature of *PIGT* deficiency. We presented five novel and two previously reported individuals [P4 and P5 (Jezela-Stanek et al., 2020)] with generalized seizures, such as myoclonic, atypical absence and tonic seizures. A detailed analysis of seizure semiology showed atypical absence seizures and bilateral tonic seizures, but it also revealed previously undetected seizure types such as myoclonic atonic seizures and eyelid-myoclonia. Myoclonic-atonic seizures are the distinguishing feature of myoclonic-atonic epilepsy (MAE), a rare epilepsy syndrome that occurs in 0.3–2.2% of children with epilepsy (Tang et al., 2020). Children with MAE usually have normal development prior to seizure onset at between 7 months and 6 years of age (Tang et al., 2020). Seizure types include myoclonic atonic, atonic, myoclonic, generalized tonic-clonic, atypical absence, and tonic seizures (Tang et al., 2020). Tang et al. (2020) showed that pathogenic genetic variants in *SYNGAP1, NEXMIF, SLC6A1, KCNA2*, *SCN2A, STX1B, KCNB1, MECP2, ASH1L, CHD4*, and *SMARCA2* are the cause of seizures in 14% of MAE individuals. Our data suggest that *PIGT* is a new candidate gene for MAE.

The eldest proband, a 29-year-old female individual with compound heterozygous *PIGT* variants (P3), exhibited action myoclonia. She first showed this symptom when she was 8 years old, and it has progressively worsened and has now become very disabling. Action myoclonia may have varying etiologies, including diseases such as Lance-Adams syndrome and the large group of progressive myoclonus epilepsies. It can be associated with epileptic EEG abnormalities (Tassinari et al., 1998), and its pathophysiological mechanisms suggest a cortical-subcortical dysfunction (Avanzini et al., 2016). It can also be associated with a cerebellar syndrome and be extremely disabling and difficult to treat, as described in individual P3. As this individual was the oldest in our cohort, we cannot dismiss the possibility that myoclonus is a later manifestation in progression of PIGT deficiency syndrome.

While 22 individuals experienced seizures, only 18 of them fulfilled the clinical diagnosis of epilepsy (Fisher et al., 2014). Apart from our individual P5, who had the His376Pro and Val528Met variants and never had seizures, we found no differences in the *PIGT* variants among individuals with and without seizures. Our study provides a detailed and in-depth description of developmental features in patients with the less severe spectrum of *PIGT* deficiency, and we also offer a glimpse into the potential natural history in adulthood. Our data are important for caregivers and health care providers when dealing with uncertainties about what to expect in the future, but also when counseling families with infants newly diagnosed with *PIGT* deficiency related to the Val528Met variant. Further investigation is needed to better understand the natural history and to elucidate the relationship between the presence of seizures and seizure semiology and the pathogenic variants in the milder spectrum of *PIGT* deficiency.

## Data Availability Statement

The original contributions presented in the study are included in the article/Supplementary Material, further inquiries can be directed to the corresponding author/s.

## Ethics Statement

The studies involving human participants were reviewed and approved by ethical committee of region Sjaelland. Written informed consent to participate in this study was provided by the participants’ legal guardian/next of kin. Written informed consent was obtained from the individual(s), and minor(s)’ legal guardian/next of kin, for the publication of any potentially identifiable images or data included in this article.

## Author Contributions

ABa and RM had the idea to write the article. ABa was responsible for collecting data from the medical records and writing the first draft of the manuscript. ABa was responsible for gathering relevant literature. GR evaluated the neurophysiological data. All authors were involved in the ongoing revision of the manuscript. All authors contributed to the revision of the final manuscript.

## Conflict of Interest

ABe was employed by the company GeneDx. The remaining authors declare that the research was conducted in the absence of any commercial or financial relationships that could be construed as a potential conflict of interest. The handling editor declared a past co-authorship with the author MG.

## Footnotes

1 https://cadd.gs.washington.edu

2 http://www.mutationtaster.org

3 https://gnomad.broadinstitute.org/

## Supplementary Material

Supplementary Table 1 Genetic overview, annotation, and classification of novel and previously published pathogenic variants in the phosphatidylinositol glycan class T protein gene.

Supplementary Video 1 Video of individual P3 displaying action myoclonia of limbs and the whole body during motor tasks and the absence of myoclonia during rest.

Supplementary Video 2 Video of individuals P11 and 12 showing motor, cognitive, and verbal capabilities.

## Abbreviations

AED: antiepileptic drugs
DD: developmental delay
EEG: electroencephalogram
ER: endoplasmatic reticulum
GPI: glycosylphosphatidylinositol
GPI-AP: glycosylphosphatidylinositol anchored protein
GPIBD: biosynthesis defects of the GPI anchor
ID: intellectual disability
MRI: magnetic resonance imaging
PIGT: phosphatidylinositol glycan class T protein
VPA: valproate.
