# Supplementary material for: Deep-Phenotyping the Less Severe Spectrum of PIGT Deficiency and Linking the Gene to Myoclonic Atonic Seizures
Source: Front Genet. 2021 May 11;12:663643. doi: 10.3389/fgene.2021.663643 (PMC8148046; doi:10.3389/fgene.2021.663643)
Supplement: Supplementary Table 1 — Genetic overview, annotation, and classification of novel and previously published pathogenic variants in the phosphatidylinositol glycan class T protein gene. [file Table_1.DOCX]

Supplementary Material

Supplementary Table 1: Genetic overview, annotation and classification of novel and previously published pathogenic variant in the phosphatidylinositol glycan class A protein gene.

| **Mutations**  **NM_015937** | **Amino acid alteration** | **Function on exon** | **SIFT**  **(D, deleterious)** | **Polyphen-2 (HVAR score) (D, probably damaging)** | **MutationTaster**  **(D, disease causing;**  **A, disease causing automatic)** | **CADD phred** | **Classification based on ACMG guidelines** | **Published** |
| --- | --- | --- | --- | --- | --- | --- | --- | --- |
| C.494-2A>G | - | Splicing | - | - | D | 26.2 | Pathogenic  ([PVS1](http://wintervar.wglab.org/mlr2.php)+PS3+[PM2](http://wintervar.wglab.org/mlr2.php)+[PM3](http://wintervar.wglab.org/mlr2.php)) | Bayat et al., 2019 (PMID: 30976099)   - Aleksandra et al., 2020 (PMID: 32725661) |
| c.769+2T>A | - | Splicing | - | - | D | 24.9 | Likely pathogenic  ([PVS1](http://wintervar.wglab.org/mlr2.php)+[PM2](http://wintervar.wglab.org/mlr2.php)+[PM3](http://wintervar.wglab.org/mlr2.php)) | Unpublished |
| c.918dupC | p.Val307ArgfsTer13 | Frameshift insertion | - | - | - | - | Pathogenic  ([PVS1](http://wintervar.wglab.org/mlr2.php)+PS3+[PM2](http://wintervar.wglab.org/mlr2.php)+[PM3](http://wintervar.wglab.org/mlr2.php)) | Lam et al., 2015 (PMID: **25943031)** |
| c.988C>T | p.Arg330Ter | Stopgain | - | - | A | 38 | Pathogenic  (PM2+PP2+PP5) | Unpublished |
| c.1096G>A | p.Gly366Arg | Missense | D | D | D | 33 | Likely pathogenic  ([PM2](http://wintervar.wglab.org/mlr2.php)+[PM3](http://wintervar.wglab.org/mlr2.php)+[PM5](http://wintervar.wglab.org/mlr2.php)+[PP3](http://wintervar.wglab.org/mlr2.php)) | Unpublished |
| c.1127A>C | p.His376Pro | Missense | D | D | D | 28.1 | VUS  (PM2+PM3) | Unpublished |
| c.1342C>T | p.Arg448Trp | Missense | D | D | D | 34 | Likely pathogenic  ([PS1](http://wintervar.wglab.org/mlr2.php)+[PM2](http://wintervar.wglab.org/mlr2.php)+[PP5](http://wintervar.wglab.org/mlr2.php)) | Unpublished |
| c.1519C>T | p.Arg507Trp | Missense | D | D | D | 35 | VUS  (PM2+PM3+PP1) | Unpublished |
| c.1520G>A | p.Arg507Gln | Missense | D | D | D | 27.1 | VUS  (PM2+PM3) | Unpublished |
| c.1580A>G | p.Asn527Ser | Missense | D | D | D | 27.1 | VUS  (PM2+PM3) | Unpublished |
| c.1582G>A | p.Val528Met | Missense | D | D | D | 34 | Pathogenic  ([PS1+PS3+PM2](http://wintervar.wglab.org/mlr2.php)+[PP3](http://wintervar.wglab.org/mlr2.php)+PP5) | Pagnamenta et al., 2017  (PMID: 28327575)  Bayat et al., 2019  (PMID: 30976099)   - Aleksandra et al., 2020 ((PMID 32725661) |
| c.1724_1725insC | p.Leu578fsTer35 | Frameshift insertion | - | - | - | - | Pathogenic  (PVS1+PS1+PS3+PM2+PP3+PP5) | Bayat et al, 2019,  PMID: 30976099 |
| c.1730dupC | p.Leu578fsTer35 | Frameshift insertion | - | - | - | - | Pathogenic  (PVS1+S1+PS3+PM2+PP3+PP5) | Pagnamenta et al., 2017 |

Abbreviation:

ACMG, American College of Medical Genetics; CADD, combined annotation dependent depletion; PolyPhen-2, polymorphism phenotyping v2; SIFT, Sorting Intolerant From Tolerant; VUS, Variant of uncertain significance.
